# Sympathetic Hyperactivity and Sleep Disorders in Individuals With Type 2 Diabetes

**DOI:** 10.3389/fendo.2019.00752

**Published:** 2019-11-01

**Authors:** Carolina López-Cano, Liliana Gutiérrez-Carrasquilla, Enric Sánchez, Jessica González, Andree Yeramian, Raquel Martí, Marta Hernández, Gonzalo Cao, Mercè Ribelles, Xavier Gómez, Silvia Barril, Ferran Barbé, Cristina Hernández, Rafael Simó, Albert Lecube

**Affiliations:** ^1^Endocrinology and Nutrition Department, Institut de Recerca Biomèdica de Lleida (IRBLleida), Hospital Universitari Arnau de Vilanova, Obesity, Diabetes and Metabolism Research Group (ODIM), Universitat de Lleida (UdL), Lleida, Spain; ^2^Respiratory Department, Institut de Recerca Biomèdica de Lleida (IRBLleida), Hospital Universitari Arnau de Vilanova-Santa María, Translational Research in Respiratory Medicine, Universitat de Lleida (UdL), Lleida, Spain; ^3^Centro de Investigación Biomédica en Red de Enfermedades Respiratorias (CIBERES), Instituto de Salud Carlos III (ISCIII), Madrid, Spain; ^4^Section of Hormones, Clinic Laboratory, Hospital Universitari Arnau de Vilanova, Universitat de Lleida (UdL), Lleida, Spain; ^5^Clinic Laboratory, Hospital Universitari Arnau de Vilanova, Universitat de Lleida (UdL), Lleida, Spain; ^6^Clinic Laboratory, Department of Laboratory Medicine, Hospital Universitari Arnau de Vilanova, Universitat de Lleida (UdL), Lleida, Spain; ^7^Diabetes and Metabolism Research Unit, Endocrinology and Nutrition Department, Vall d'Hebron Institut de Recerca (VHIR), Hospital Universitari Vall d'Hebron, Universitat Autònoma de Barcelona, Barcelona, Spain; ^8^Centro de Investigación en Red en Diabetes y Enfermedades Metabólicas Asociadas (CIBERDEM), Instituto de Salud Carlos III (ISCIII), Madrid, Spain

**Keywords:** type 2 diabetes, sympathetic hyperactivity, sleep apnea-hypopnea syndrome, sleep breathing, cardiovascular risk

## Abstract

**Introduction:** Many studies on the impact of type 2 diabetes mellitus (T2DM) on sleep breathing have shown a higher prevalence and severity of sleep apnea-hypopnea syndrome (SAHS) in those with T2DM. Moreover, an increased activity of the sympathetic nervous system has been described in both pathologies. This cross-sectional study aimed to assess sympathetic activity in patients with T2DM, and to investigate the relationship between sympathetic activity and polysomnographic parameters.

**Materials and Methods:** Thirty-six patients with T2DM without known clinical macrovascular nor pulmonary disease and 11 controls underwent respiratory polygraphy, and their cardiac variability and 24-h urine total metanephrines were measured.

**Results:** SAHS was highly prevalent with a mean apnea-hypopnea index (AHI) in the range of moderate SAHS. In patients with T2DM, the nocturnal concentration of total metanephrines in urine were higher than diurnal levels [247.0 (120.0–1375.0) vs. 210.0 (92.0–670.0), *p* = 0.039]. The nocturnal total metanephrine concentration was positively and significantly associatedwith the percentage of sleeping time spent with oxygen saturation <90%(CT90). In the entire population and in subjects with T2DM, the multivariate regression analysis showed a direct interaction between the nocturnal concentration of urine metanephrines and the CT90.

**Conclusion:** These findings suggest that the increase in sympathetic activity previously described in patients with T2DM could be mediated through nocturnal breathing disturbances. The diagnosis and treatment of SAHS may influence sympathetic activity disorders and may contribute to an improvement in T2DM and cardiovascular risk.

## Introduction

A wealth of clinical and cross-sectional studies showed a higher prevalence of sleep apnoea-hypopnea syndrome (SAHS) in patients with type 2 diabetes (T2DM). The data vary according to the population studied, but reach a prevalence of up to 85%, with 70% for the range of moderate SAHS (1–5). Despite the lack of prospective studies to demonstrate the SAHS impact in patients with T2D, it is well known that intermittent nocturnal hypoxia is associated with poorer glycaemic control and an increase in cardiovascular risk (6–13). Alternatively, patients with T2D, in comparison with the general population have a negative impact on breathing during sleep, creating greater sleep fragmentation, an increase in micro-awakenings and a higher prevalence of nocturnal hypoxia episodes (14–18). However, there is little evidence and some controversy on whether these alterations are responsible for hyperactivation of the sympathetic nervous system described in patients with T2DM.

Usually, the autonomic nervous system shows episodic oscillations of broad-spectrum activity during sleep with dynamic fluctuation of vital factors such as heart rate, blood pressure and ventilation (19, 20). Moreover, changes in sleep architecture are associated with increased sympathetic activity, leading to endothelial dysfunction and decreased nitric oxide concentration (21–23). In this way, episodes of nocturnal hypoxia also activate the sympathetic system, promoting alterations in cardiac variability and probably endothelial damage, which contributes to increased cardiovascular risk (24–27). Sympathetic overactivity has also been associated with metabolic dysfunction such as insulin resistance, obesity, hypertension and dyslipidaemia (28–30). Even genetic polymorphisms have been identified the sympathetic nervous system of humans and animals and seem to contribute to the development of obesity and T2DM in certain populations (31–33).

Since the origin of sympathetic hyperactivation in patients with T2DM is not precisely known, we propose to analyse the relation between the presence of breathing disorders that characterize their sleep with data related with sympathetic activity. We aimed to deepen this hypothesis by studying the polysomnographic record of 36 subjects with T2DM and its relationship with measurement of urine metanephrine concentration, heart rate variability (HRV) and other serum markers of sympathetic activity.

## Materials and Methods

### Ethic Statement

The study was approved by the human ethics committee from the Arnau de Vilanova University Hospital and was conducted according to the ethical guidelines of the Helsinki Declaration. Informed written consent was obtained from all participants included in the study.

### Description of the Study Population

In this cross-sectional study, a total of 36 patients of Caucasian origin with T2DM were prospectively recruited between September 2017 and July 2018 at the outpatient clinic in the Endocrinology Department of the Arnau de Vilanova University Hospital. Willing participants were given detailed information about the objectives and procedures of the study.

Participants fulfilled the following inclusion criteria: T2DM with more than 5 years of known evolution, men and women aged 40 and 70 years without any history of vascular disease or lung disease and a BMI of less than 40 kg/m^2^. The exclusion criteria were presence of type 1 diabetes, chronic kidney disease, active neoplasia, neuromuscular diseases, active smoking or former smokers, history of alcohol abuse and treatment with continuous positive airway pressure (CPAP). Patients who were treated with drugs with activity on the central nervous system (e.g., hypnotics, antidepressants, sedatives, psycholeptics) were also excluded from the study. No pregnant women were included. A control group of 11 healthy subjects without T2DM or lung disease and well matched by age, body mass index (BMI), neck and waist circumferences and blood pressure was also recruited during the same period among the relatives of patients with diabetes, as well as the employees of our institution.

### Measurement of Sleep-Disordered Breathing

The 36 recruited patients and the 11 controls underwent a non-attended respiratory polygraphic record with a portable Sleep Diagnostic Alice PDx (Philips Respironics, Spain) which records nasal airflow (nasal cannula), respiratory effort (chest and abdominal bands), snoring, body position and finger pulse oximetry. The same technician manually checked all sleep studies to reduce variability. Records with less than 5 h of correct signal were repeated. An apnea was defined as cessation of airflow for more than 10 s. Hypopnea was defined as a reduction in respiratory signals for at least 10 s associated with oxygen desaturation of 4% or more. The apnea-hypopnea index (AHI) was calculated as the total number of respiratory events (apneas plus hypopneas) divided by the recording time in bed (per hour of sleep). On this basis, SAHS was defined as an AHI >10 events/hour (e/h). The cumulative percentage of time spent with oxygen saturations below 90% (CT90) was also assessed (34–36).

### Laboratory Assessment

After patients fasted overnight for 12 h, venous blood was collected from the antecubital vein. Samples were separated by centrifugation (2,000 g at 4°C for 20 min) and analyzed in the clinical laboratory of our hospital using standard methods to obtain biochemical parameters. The measurement of urine metanephrines was realized by high performance liquid chromatography. In order to compare the concentrations of urine metanephrines between day and night, urine collection was carried out in two 12-h time periods [from 8:00 to 20:00 (day) and from 20:00 to 8:00 (night)]. The serum concentration of hormones related with glucose metabolism [cortisol, insulin, glucagon, insulin-like growth factor-1 (IGF-1) and growth hormone (GH)] were also determined through chemiluminescence enzyme immunoassay. All samples were collected in the absence of clinical evidence of active infection or inflammatory diseases.

### Cardiac Variability Study: Heart Rate Variability Parameters

Heart rate variability (HRV), defined as the measurement of the variation in milliseconds between heart beats, was studied in its R-R intervals, through a QHRV device with DT-HW6c hardware (QHRV, Santa Bárbara, CA, United States). The results were analyzed after measuring in four different stages: after a 15-min rest, breathing deeply, in Valsalva and in a standing position. In the time domain, we analyzed RR intervals, standard deviations of RR intervals (SDNN), the square root of the mean squared difference of successive RR intervals (RMSSD), and the percentage of adjacent NN intervals differing by more than 50 ms (pNN50). This last measurement was referred to as “short-term” HRV and reflects the parasympathetic influence on the heart rate. In the frequency domain, we analyzed the low-frequency band (LF, an index of both sympathetic and parasympathetic activity), high-frequency band (HF) and very low-frequency (VLF, that reflects thermoregulatory mechanisms, fluctuation in activity of the renin–angiotensin system, and the function of peripheral chemoreceptors) (37). Patients were advised not to consume food, coffee or nicotine 3 h before the test and to not receive treatment with anticholinergics, fludrocortisone, antihistamines, alpha or beta antagonists, or anxiolytics during 48 h before the test.

### Statistical Analysis

Normal distribution of the variables was assessed using the Kolmogorov-Smirnov test. Data were expressed as the mean ± standard deviation or percentage. Given their skewed distribution, AHI, CT90 and serum triglycerides are shown as median (range) and were logarithmically transformed to achieve a normal distribution. The relationship between the continuous variables was examined by the Pearson linear correlation test. Univariate and multivariate logistic regression models were computed. A stepwise multivariate regression analysis was performed to explore the variables independently related to nocturnal urine metanephrines. Variables significantly associated with changes in urine metanephrines in the bivariate analysis (i.e., CT90 and serum glucagon concentration), together with heart rate variability parameters and clinically relevant variables (i.e., gender, BMI, age, smoking habit, blood pressure, AHI, neck circumference and HbA1c) were included in the analysis. All *p*-values were based on a two-sided test of statistical significance. Significance was accepted at the level of *p* < 0.05. Statistical analyses were performed using the SPSS statistical package (IBM SPSS, Statistics for Windows, Version 20.0. Armonk, NY, USA).

## Results

The main clinical features and metabolic data of the study population are presented in Table 1. Briefly, patients with T2DM were 56.3 ± 8.7 years old, mainly female (27.7%) and obese (BMI 31.9 ± 4.8 kg/m^2^) and showed a poor metabolic control (HbA1c 8.0 ± 1.3%). Regarding measurements from the non-attended polygraphy, they exhibited a median AHI of 23.5 (3.0–78.0) e/h, with a median CT90 of 7.5 (0–64)%. Finally, hormone values and HRV parameters were within the normal range (Table 2).

**Table 1:** Baseline main clinical, metabolic and sleep-breathing characteristics of participants in the study.

	**Type 2 diabetes**	**Control group**	***p***
N	36	11	–
Age (years)	56.3 ± 8.7	51.2 ± 8.8	0.101
Women, n (%)	10 (27.7)	11 (100%)	<0.001
BMI (Kg/m^2^)	31.9 ± 4.8	32.5 ± 5.8	0.745
Neck circumference (cm)	41.9 ± 4.2	39.6 ± 2.8	0.095
Waist circumference (cm)	113.0 ± 11.0	116.0 ± 10.8	0.431
HbA1c (%)	8.0 ± 1.3	5.5 ± 0.5	<0.001
LDL Cholesterol (mmol/l)	2.8 ± 1.0	3.2 ± 1.1	0.337
Triglycerides (mmol/l)	1.7 (0.8–3.7)	1.1 (0.8–2.0)	0.089
Systolic blood pressure (mm Hg)	131.6 ± 12.6	131.9 ± 10.5	0.902
Diastolic blood pressure (mm Hg)	77.6 ± 8.2	80.7 ± 5.3	0.260
AHI (events/hour)	23.5 (3.0–78.0)	14.0 (0.0–67.0)	0.227
CT90 (%)	7.5 (0.0–64.0)	6.5 (0.0–44.0)	0.355

*Data are mean ± SD, median (range) or n (percentage). BMI, body mass index; HbA1c, glycated hemoglobin; LDL, low density lipoprotein; AHI, apnea-hypopnea index; CT90, percentage of time spent with oxygen saturations below 90%*.

**Table 2:** Hormones values and heart variability parameters of participants in the study.

	**Type 2 diabetes**	**Control group**	***p***
Cortisol (nmol/L)	330.0 (41.0–616.2)	452 (211.3–798.3)	0.094
Insulin (mIU/L)	100.3 (24.0–523.8)	128.5 (40.1–188.0)	0.899
IGF-1 (ng/mL)	135.1 (55.3–273.2)	149.9 (85.0–215.9)	0.461
Glucagon (pg/mL)	136.0 (61.0–304.0)	112.0 (63.0–183.0)	0.348
GH (ng/mL)	0.1 (0.0–2.1)	0.1 (0.0–1.2)	0.800
LFa/RFa resting	0.9 (0.3–2.0)	0.7 (0.2–1.8)	0.299
LFa/RFa respiratory	0.6 (0.2–1.7)	0.4 (0.3–0.7)	0.099
LFa/RFa valsalva	1.7 (0.8–3.0)	1.7 (0.6–3.5)	0.591
LFa/RFa standing	1.1 (0.3–2.8)	0.7 (0.5–2.2)	0.022
SDNN	21.5 (7.1–85.3)	39.5 (17.4–76.7)	0.013

*Data are median (range). IGF-1, insulin-like growth factor-1; GH, growth hormone; LFa, low frequency component; RFa, high frequency component; SDNN, standard deviation of beat to beat or NN intervals*.

Urine concentrations of total metanephrines were statistically higher during the night compared with the concentrations during the day period in the group of subjects with T2DM [247.0 (120.0.1375.0) vs. 210.0 (92.0–670.0), *p* = 0.039]. However, no differences between day and night were observed in the control group [243.0 (104.0–560.0) vs. 250.5 (94.0–485.0), *p* = 0.674).

**Figure 1:**
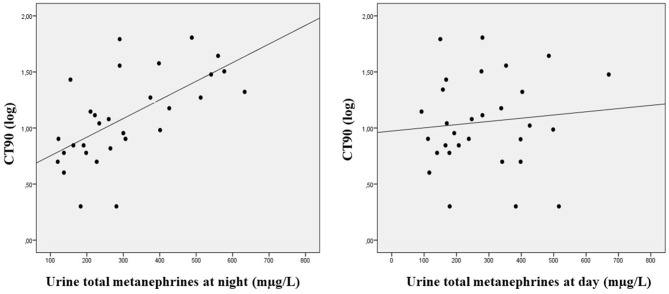
Linear correlation between concentration of urine metanephrines and percentage of time with oxygen saturations below 90%.

Linear correlations between day and night metanephrines and serum hormones and heart rate variability parameters are shown in Table 3. Whereas the nocturnal concentration of urine metanephrines were positively associated with values of CT90 (*r* = 0.617, *p* < 0.001), this association disappeared with the metanephrines collected during the day (*r* = 0.093, *p* = 0.644) (Figure 1). In addition, nocturnal and daily urine metanephrines were negatively associated with serum glucagon concentrations (*r* = −0.427, *p* = 0.008). In the multivariate regression analysis including the entire population we found a significant direct interaction between the concentration of urine metanephrines at night and the percentage of time spent with oxygen saturation <90% (*p* = 0.001) [together with serum glucagon concentration (*p* = 0.001), LFa/RFa resting (*p* = 0.009) and diastolic blood pressure (*p* = 0.047), *R*^2^ = 0.741]. This indicated that the increase in the secretion of metanephrines at night could be related with the severity of nocturnal hypoxia (Table 4). When subjects with T2DM were assessed alone, serum glucagon concentrations (*p* = 0.011) and CT90 (*p* = 0.016) still independently predicted nocturnal urine metanephrines (*R*^2^ = 0.549). However, in the control group, only diastolic blood pressure and serum glucagon concentrations but not CT90 were significantly related with nocturnal total metanephrines.

**Table 3:** Linear correlations between day and night metanephrines and serum hormones and heart rate variability parameters.

	**Night metanephrines**		**Day metanephrines**	
	***r***	***p***	***r***	***p***
CT90 (log)	0.617	<0.001	0.093	0.644
AHI (log)	0.146	0.356	0.426	0.146
Cortisol (mmol/L)	0.069	0.677	−0.102	0.522
Insulin (mIU/L)	0.235	0.145	0.050	0.752
IGF-1 (ng/mL)	0.045	0.784	0.039	0.802
Glucagon (pg/mL)	−0.427	0.008	−0.452	0.003
GH (ng/mL)	0.231	0.151	0.101	0.519
LFa/RFa respiratory	−0.163	0.335	−0.176	0.271
LFa/RFa valsalva	0.085	0.612	0.236	0.132
LFa/RFa standing	0.127	0.448	0.035	0.828
LFa/RFa resting	−0.161	0.334	−0.137	0.386

*IGF-1, insulin-like growth factor-1; GH, growth hormone; LFa, low frequency component; RFa, high frequency component*.

**Table 4:** Variables independently related to nocturnal urine total metanephrines in the multiple regression analysis (stepwise method) in the entire population.

	**β**	**Beta (95% IC)**	***p***
CT90 (log)	0.520	191.2 (93.9 to 288.6)	0.001
Glucagon (pg/mL)	−0.505	−1.3 (−2.0 to −0.6)	0.001
LFa/RFa resting	−0.379	−148.9 (−255.4 to −42.5)	0.009
Systolic blood pressure (mm Hg)	0.277	6.0 (0.0 to 12.0)	0.047
AHI (events/hour)	−0.212	–	0.095
Smoking status ^*^	−0.240	–	0.114
GH (ng/mL)	−0.260	–	0.172
LFa/RFa standing	−0.218	–	0.196
Age (years)	−0.148	–	0.289
IGF-1 (ng/mL)	0.138	–	0.356
Insulin (mIU/L)	0.132	–	0.370
Systolic blood pressure (mmHg)	−0.246	–	0.371
Cortisol (mmol/L)	−0.115	–	0.434
LFa/RFa respiratory	−0.100	–	0.436
HbA1c (%)	0.120	–	0.443
BMI (kg/m^2^)	0.074	–	0.596
Gender (men/women)	−0.047	–	0.742
LFa/RFa respiratory	−0.041	–	0.763
LFa/RFa Valsalva	0.026	–	0.866
Neck circumference (cms)	−0.016	–	0.914
Constant	–	−58.1 (−540.7 to 424.3)	0.802
*R*^2^ = 0.741			

*CT90, percentage of time spent with oxygen saturation below 90%; LFa, low frequency component; RFa, high frequency component; AHI, apnea-hypopnea index; GH, growth hormone; IGF-1, insulin-like growth factor-1; HbA1c, glycated hemoglobin; BMI, body mass index*.

## Discussion

The main findings in this study were that a subgroup of patients with T2DM exhibited an increase in urine metanephrines at night associated with nocturnal hypoxia and with a decrease in serum glucagon concentrations, specifically for the parasympathetic tone. This autonomic disturbance can be explained by the deleterious effects of diabetes during night-time sleep, leading to an important nocturnal hypoxia.

It is well known that T2DM patients exhibit a hyperactivation of the sympathetic nervous system (SNS) that amplifies the effect of hyperglycaemia, increasing both cardiovascular risk and microangiopathic diabetic complications (38). The origin of this sympathetic hyperactivation has not been precisely described. We propose that, both intermittent hypoxia and the greater number of micro-awakenings that characterize the sleep of T2DM patients could explain the increase in SNS activity.

While the evidence is controversial, previous studies have demonstrated that apnea and hypopnea events are accompanied by concomitant cyclic variations in heart rate and changes in both sympathetic and parasympathetic activity without differences in subjects with diabetes (39, 40). As far as we know, hyperactivation of the autonomic system has been described in patients with T2DM but with involvement of both patterns (sympathetic and parasympathetic) and it was related to male sex and the presence of neuropathy (41–43). None of our patients had clinical neuropathy, and the test group was predominantly female.

Although we cannot elucidate whether the effects of hypoxia and diabetes overlap with the effects of obesity, some recent studies have shown an increased SAHS prevalence in non-overweight patients with Type 1 diabetes, suggesting that nocturnal hypoxia might be more related to autonomic disturbances rather than obesity (44, 45).

Our study combined data from men and women in the analyses, but differences regarding sex and menopausal status have been previously described. In this way, some findings suggest that men have a higher sympathetic nerve activity than women after the development of essential hypertension, and that this could have implications for gender-specific management of hypertension (46, 47). Similarly, muscle sympathetic nerve activity has been described to be greater in postmenopausal women in comparison with premenopausal women (48). In our study, the LFa/RFa respiratory parameter was the only parameter related with heart rate variability that was significantly decreased in women when compared to men [0.7 (0.2–1.7) vs. 0.4 (0.2–0.8), *p* = 0.002], and it was also the only parameter that was positively associated with age in the bivariate analysis (*r* = 0.318, *p* = 0.038). The other heart rate variability parameters and serum hormone concentrations showed no differences between genders nor correlations with age. In addition, gender and age were not independently associated with total nocturnal urine metanephrines in the multivariate analysis.

The present study does not have sufficient power to exhibit endothelial damage or cardiovascular risk caused by this hyperactivity, but some previous studies have described the relationship between variability in autonomic function and an increase in insulin resistance, oxidative stress and inflammatory response that cause endothelial celldamage, development of atherosclerosis and increased cardiovascular risk (49–51). Along the same lines, it has been described that cardiovascular autonomic neuropathy contributes to a poorer prognosis in ischemic heart disease and heart failure (52). Also, the decrease in HRV can predict the progression of atherosclerosis in T2DM patients (53).

Our study has some limitations. First, this is a cross sectional whereby causality cannot be determined. The size of the study might have been inadequate, and a prospective study with a higher number of patients could yield better results. In addition, it should be noted that the control group in our study was composed only of women. As previously mentioned, women have a lower sympathetic nerve activity than men, so this data could influence our results. However, in the multivariate regression analysis, gender was not related to the concentration of urine metanephrines at night. Second, the HRV study might have been influenced by differences in baseline physical condition or cardiac status. We did not perform any cardiac evaluation although all participants were asked for cardiovascular diseases and arrhythmias and neither of them were athletic. Third, the effects of the antidiabetics (insulin, SGLT2, incretins) could not be excluded.

## Conclusion

We suggest that the increased sympathetic activity previously described in patients with T2DM is mediated through the deleterious effect of diabetes in nocturnal breathing. In addition, sympathetic activity is associated with disorders of autonomic tone at resting, suggesting a new pathological pathway between T2DM and cardiovascular risk. More studies are needed to clarify whether treatment of SAHS will improve sympathetic hyperactivity, T2DM and cardiovascular outcomes in these patients.

## Data Availability Statement

The datasets generated for this study are available on request to the corresponding author.

## Author Contributions

CL-C, LG-C, and ES recruited patients, collected and analyzed data, wrote the first draft of the manuscript, and had final approval of the version for publication. FB supervised the research, interpreted data, and critically reviewed the draft of the article. JG, SB, ES, MR, and RM collected and analyzed data, and critically reviewed the draft of the article. LG-C, MH, and XG recruited patients, collected data, and contributed to the discussion. GC, MR, and AY collected and analyzed data, and contributed to the discussion. CH and RS designed the study, supervised the statistical analysis, interpreted data, critically revised draft of the article, and had final approval of the version for publication. CL-C and AL designed the study, supervised the research, analyzed and interpreted data, and wrote the manuscript.

### Conflict of Interest

The authors declare that the research was conducted in the absence of any commercial or financial relationships that could be construed as a potential conflict of interest.
